# New Insights in the Mobilization of Hematopoietic Stem Cells in Lymphoma and Multiple Myeloma Patients

**DOI:** 10.1155/2014/835138

**Published:** 2014-08-14

**Authors:** Maria K. Angelopoulou, Pantelis Tsirkinidis, Georgios Boutsikas, Theodoros P. Vassilakopoulos, Panayiotis Tsirigotis

**Affiliations:** ^1^Department of Hematology and Bone Marrow Transplantation, Laikon General Hospital, National and Kapodistrian University of Athens, 17 AgiouThoma, Goudi, 11527 Athens, Greece; ^2^Department of Hematology, 401 Army Forces Hospital, 138 Mesogeion Avenue, 11525 Athens, Greece; ^3^2nd Propedeutic Department of Internal Medicine, National and Kapodistrian University of Athens, 1 Rimini Street, Chaidari, 12462 Athens, Greece

## Abstract

Following chemotherapy and/or the administration of growth factors, such as granulocyte-colony stimulated factor (G-CSF), hematopoietic stem cells (HSC) mobilize from bone marrow to peripheral blood. This review aims to systematically present the structure of the HSC “niche” and elucidate the mechanisms of their mobilization. However, this field is constantly evolving and new pathways and molecules have been shown to contribute to the mobilization process. Understanding the importance and the possible primary pathophysiologic role of each pathway is rather difficult, since they share various overlapping components. The primary initiating event for the mobilization of HSC is chemotherapy-induced endogenous G-CSF production or exogenous G-CSF administration. G-CSF induces proliferation and expansion of the myelomonocytic series, which leads to proteolytic enzyme activation. These enzymes result in disruption of various receptor-ligand bonds, which leads to the disanchorage of HSC from the bone marrow stroma. In everyday clinical practice, CXC chemokine receptor-4 (CXCR4) antagonists are now being used as mobilization agents in order to improve HSC collection. Furthermore, based on the proposed mechanisms of HSC mobilization, novel mobilizing agents have been developed and are currently evaluated in preclinical and clinical studies.

## 1. Introduction

Autologous hematopoietic stem cell transplantation (ASCT) is a widely used therapeutic strategy in the treatment of multiple myeloma and relapsed/refractory lymphomas. It can offer long-term disease control or even cure in a substantial proportion of patients. The prerequisite of ASCT is a successful and adequate stem cell mobilization and collection. Initial observations regarding the steady state circulation of hematopoietic stem cells (HSC) in the blood led to the study of HSC kinetics after the administration of chemotherapy with or without growth factors. Thus, nowadays, collection of HSC from the bone marrow (BM) has been neglected at least in the autologous transplantation setting and has been largely replaced by peripheral blood stem cell collection through cell separators. Patients who fail to collect ≥2.0 × 10^6^ CD34^+^ stem cells/kg of body weight cannot undergo ASCT and thus experience its benefits. The deep knowledge and understanding of HSC mobilization will give insight into the mechanisms of “poor mobilization” and moreover may help in developing new mobilizing agents.

## 2. The Stem Cell Niche

The term HSC was introduced for the first time by Alexander Maximov in 1909 [1]. HSC are primitive undifferentiated cells capable of giving rise to all mature cells of the hematopoietic system through proliferation, differentiation, and maturation. Moreover, they have a self-renewal capacity and the balance between their quiescence and proliferative potential is under strict control. This in part succeeded through asymmetrical cell division. One HSC gives rise to 2 daughter cells, one of which remains as a pluripotent stem cell and the other migrates to the main bone marrow compartment, where it differentiates to its progenies [2, 3]. The specialized environment, where this fine balance is maintained, is described as the “stem cell niche” and was introduced by Ray Schofield in 1978 [4].

Anatomically, the niche is located in close proximity to the endosteum and is supported by a variety of cells and molecules referred as “stroma.” The main representatives of the stroma are bone tissue cells (osteoblasts (OB), osteoclasts (OC), osteomacrophages (OMAC), chondrocytes, fibroblasts, and fat cells), reticuloendothelial cells (dendritic cells, lymphocytes, and macrophages), endothelial cells, as well as mesenchymal stem cells (MSC), myocytes, and cells of the autonomous nervous system. Noncellular stromal elements include the extracellular matrix (ECM), collagen, and minerals [5].

Three types of niches have been recognized: the endosteal (osteoblastic), the reticular, and the vascular (endothelial). The former is located at the endosteum and consists mainly of the “spindle-shaped N-cadherin^+^CD45^−^ osteoblastic cells” (SNO) [5]. The SNO are supported by the OMAC [6, 7]. The reticular niche is diffusely developed in the BM as a “data network” and consists of specialized reticuloendothelial cells, called “CXCL12-abundant reticular cells” (CAR), which are in close contact with immune cells (B-lymphocytes, plasma cells, plasmacytoid dendritic cells, and NK-lymphocytes), sinusoidal endothelial cells, and Nestin+ MSC^8^. The third niche type refers to a microenvironment rich in oxygen, with low calcium content, consisting of the vascular sinusoidal endothelial cells. Among them, the BM-derived endothelial cells (BMEC) are in close proximity to Nes+ MSC and CAR [7–10]. HSC represent 0.005% of all BM cells, while the multipotent progenitors (MPP) are approximately 0.1%. Human HSC are CD34^+^, CD38^−^, CD45RA^−^, and CD90^+^. However the ultimate proof of their “stemness” comes from experimental in vivo assays, such as long-term repopulating (LTRA), competitive repopulation unit (CRU), SCID repopulating cell (SRC), and limiting dilution assays [11].

## 3. Mechanisms of Quiescence and Self-Protection of HSC

The stem cell niche is essential for the quiescence of HSC. More than 70% of them are in the G_0_ phase of the cell cycle, while only 10% of their progenies are quiescent. It has been shown that approximately 30% of the quiescent HSC divide every 145–193 days, while a more active subpopulation does so every 28–36 days [12]. These two different subpopulations represent the long-term HSC (LT-HSC), capable of sustaining life-long hematopoiesis, and the short-term HSC (ST-HSC), giving growth to hematopoiesis lasting for several weeks, respectively.

LT-HSC protect themselves from DNA damage by limiting the number of their cellular divisions. The main DNA-repair mechanism of HSC is the nonhomologous end joining, NHEJ [13]. In addition, HSC are capable of transporting damaging agents outside of the cell by ATP-dependent cell transporters, such as BCRP-1 [14]. A variety of other mechanisms contributing to their quiescence are active, such as molecular regulation of the cell cycle through cyclins, cyclin-dependent kinases (CDK), and CDK inhibitors, signaling through thrombopoietin and transforming growth factor *β*, the ubiquitin/proteasome pathway, and signaling pathways through ATM, P13K-Akt, mTOR, Mdm2/p53, osteopontin, and Notch and Wnt [15–21]. In addition, the hypoxic microenvironment of the BM compared to blood is thought to play a role in the balance between quiescence and differentiation of HSC [22, 23].

It is obvious that the description of the aforementioned three types of niches is undertaken for understanding purposes. They do not represent different anatomical compartments, since bone and vessels are in close proximity to each other [23]. The “niche” is an integrated specialized microenvironment unit that aims to sustain a stable number of HSC at any time, allowing only for a minimal release of HSC to the circulation under steady state conditions. Under “stress,” like after recovery from chemotherapy, a massive release of HSC into the blood is observed. This phenomenon may last from hours to few days and is described as HSC mobilization. The underlying mechanisms of chemotherapy/growth factor-induced mobilization have been extensively studied. However, it is not known, whether the same mechanisms are active in steady state mobilization [23, 24]. Due to the wide application of HSC transplantation in every day clinical practice, it is important to get a thorough insight in the mechanisms of HSC mobilization.

## 4. Mechanisms of HSC Mobilization

A concise presentation of the main cells, receptors, and ligands that interact with each other during the process of mobilization is shown in Table 1. Most of the data regarding mechanisms of HSC mobilization come either from in vitro or from in vivo animal studies; thus it is uncertain whether the exact same mechanisms are functional in the human organism. Moreover, several pathways described thereafter are overlapping and many aspects of this complex cascade of events are still obscure. Mobilization of HSC involves an active interplay between cytokines, chemokines, adhesion molecules, and proteolytic enzymes, leading to the breakdown of HSC anchorage on their niches and their subsequent egress to the blood.

In 1976 Richman et al. described an increase of HSC in the blood of patients who had undergone chemotherapy [25]. Later, a similar increase was observed after the administration of recombinant growth factors [26]. Sequentially, it was proven that both granulocyte-colony stimulating factor (G-CSF) and chemotherapy result in HSC mobilization through the same mechanisms, since chemotherapy leads to endogenous G-CSF increase [27]. Thus the main initiating event of HSC mobilization is either exogenous or endogenous G-CSF or the combination of both.

### 4.1. G-CSF

G-CSF promotes proliferation and maturation of the myeloid series, while at the same time it induces substantial changes in the bone marrow stroma, leading to the rise of HSC in the circulation by 60 times compared to baseline [28]. Only cells of the myelomonocytic series, including macrophages and OMAC, express the G-CSF receptor (CD114), whereas HSC do not [29]. Thus mobilization through G-CSF is indirect, through the following proposed pathways.

- It directly activates OMAC and macrophages, a fact that downregulates neighboring SNO, Nes+ MSC, and CAR, leading to reduced production of stromal-derived factor-1 (SDF-1) by these latter cells [7, 30]. Reduced SDF-1 leads in turn to loosening of the SDF-1/CXCR4 bond of HSC on BM stroma.
- The hyperplastic myelomonocytic series (through G-CSF) secrete a large variety of proteases, which induce proteolytic cleavage/clearance of SDF-1, leading to the release of the CXCR4 receptors on HSC and their subsequent liberation from the BM stroma. The activity of the proteases is further assisted by the cleavage of protease inhibitors [28]. The most widely studied protease is metalloproteinase-9 (MMP-9) [31]. However the dipeptidase CD26 may be more important, since it inactivates SDF-1. Moreover, CD26−/− mice display impaired mobilization [32]. Other proteases implicated in this process are cathepsins G and K and neutrophil elastase [33]. Mobilization through G-CSF is associated with a decrease in heparanase levels and a concomitant increase of MMP-9 and cathepsins [34]. The exact role of other proteins, such as complement [35] and the fibrinolysis/plasminogen [36] system, has not been elucidated yet.
- The same proteolytic G-CSF-induced mechanism is responsible for the degradation of VCAM-1, fibronectin, and OPN, leading to reduced cellular adhesion of HSC through their receptor VLA-4 to BM stroma [28, 37].
- G-CSF evokes a shift of HSC to more central locations in BM, close to the vascular endothelium due to increased oxygenation needs of the HSC [38]. HSC move towards higher oxygen concentrations, a fact that precedes their migration to the periphery.
- G-CSF reacts as a potent noradrenalin reuptake inhibitor. An additive effect of G-CSF and tricyclic antidepressants in HSC mobilization has been shown [39]. Vice versa chemical sympathectomy with beta blockers results in impaired HSC mobilization in mice [40]. Moreover, the sympathetic nervous system results in reduced SDF-1 production and directly reacts with HSC, since the latter express beta-adrenergic and dopaminergic receptors, which activate after G-CSF administration through Wnt signaling [41]. In addition, beta-2 adrenergic signaling augments the expression of vitamin D receptors on SNO, a crucial event for G-CSF mobilization [42].

The two basic G-CSF-induced mechanisms of SDF-1/CXCR4 and VCAM-1/VLA4 disruption have a synergistic effect. Coadministration of G-CSF and CXCR4 inhibitors or G-CSF and anti-VLA4 antibodies results in additive and more potent HSC mobilization compared to G-CSF alone [28].

### 4.2. SDF-1/CXCR4

SDF-1 (CXCL12) is a CXC chemokine, secreted by various BM stromal cells, such as CAR, Nes+ MSC, osteoblasts, and endothelial cells. The interaction of SDF-1 with its receptor CXCR4 on HSC plays a key role in HSC retention and trafficking. The expression of CXCR-4 on HSC is enhanced through a signaling cascade involving cAMP, phosphatidylinositol-4,5-biphosphate 3 kinase (PI3K), a number of GTPases, and atypical protein kinase C isoform *ζ* (PKC-*ζ*) [43]. PKC*ζ* induces motility, adhesion and survival of CD34^+^ cells and MMP-2, and MMP-9 secretion. In addition, CXCR4 expression is dependent on stem cell factor (SCF) [44]. SDF-1 is the most powerful HSC chemoattractant and survival regulator. Peled et al. were among the pioneers investigating the crucial role of SDF-1/CXCR4 interaction in HSC homing. They showed that treatment of human cells with a CXCR-4 antibody prevented their engraftment in a xenotransplantation model [44]. As already emphasized, the disruption of the SDF-1/CXCR4 axis represents the major mechanism by which HSC are released from their niche. This event is initiated by exogenous or endogenous G-CSF. The ultimate proof of the pivotal role of SDF-1/CXCR-4 axis has been shown from experiments, where HSC mobilization was inhibited by neutralizing anti-SDF-1 or anti-CXCR-4 antibodies [33].

The recently introduced mobilizing agent CXCR4 inhibitor plerixafor (AMD3100) disrupts the SDF-1/CXCR4 axis in a synergistic way to G-CSF. The following hypotheses have been proposed for its mechanism of action [45]. (a) The inhibition of CXCR4 on HSC leads to loss of their sensitivity to SDF-1. As a result, HSC are attracted to the circulation through a positive signal, most likely the sphingolipid sphingosine-1-phosphate (S1P). Alternatively HSC passively move to the blood. (b) Plerixafor targets SDF-1 that is produced by BM stromal cells, causing a decrease of its levels and leading ultimately to mobilization. Only a minor to moderate decrease in SDF-1 levels is sufficient for the migration of HSC from BM to blood. (c) Bonig and Papayannopoulou have suggested that plerixafor does not actually cause mobilization from the BM, but it traps HSC in the circulation by binding on CXCR4 and leading to loss of chemoattraction to SDF-1. This hypothesis requires a rapid steady state HSC turnover between marrow and other tissues and may explain the high HSC numbers observed in the blood within 30 minutes after plerixafor administration [23].

Comparing the differences between G-CSF- and plerixafor-induced mobilization, it should be noted that CXCR4 antagonists lead to a more rapid mobilization. Additionally, CXCR4 antagonists do not induce either myeloid hyperplasia or proteolysis. It has been shown from experiments in primates that myeloid hyperplasia is not necessary for mobilization [27]. Recent studies have evaluated possible differences in cell composition between G-CSF and plerixafor-mobilized grafts. Plerixafor, G-CSF, and plerixafor plus G-CSF mobilize different types of CD34^+^ cells. The proportion of the most primitive HSC (CD34^+^ CD133^+^ CD38^−^) has been reported higher in grafts from patients with lymphomas mobilized with chemotherapy/G-CSF plus plerixafor [46]. Fruehauf et al. found increased expression of several antiapoptosis, cell cycle, DNA repair, cell motility, and oxygen transport genes in plerixafor plus G-CSF-mobilized CD34^+^ cells [47]. Donahue et al. studied HSC collected from primates and show that plerixafor plus G-CSF mobilizes a greater proportion of B- and T-cell precursors than either G-CSF or plerixafor alone. Moreover they suggest that G-CSF-mobilized CD34^+^ cells may contain a wider range of lineage progenitors and hypothesize that CD34^+^ cells mobilized with G-CSF may result in faster neutrophil recovery, while those mobilized with plerixafor or plerixafor plus G-CSF may have more rapid B-cell and T-cell recovery [48]. Prospective studies focusing on the reconstitution of different cellular subpopulations are needed in order to draw firm conclusions. Regarding, other cellular populations, plerixafor-mobilized grafts have increased absolute numbers of T cells, helper T- and suppressor T-cell subsets, as well as interferon-gamma and tumor necrosis factor-alpha secreting T-lymphocytes compared to G-CSF-mobilized grafts [46, 49].

### 4.3. Sphingolipids, Prostaglandins, and Eicosanoids

The SDF-1/CXCR4 axis seems to be influenced by sphingolipids, with the main representative being sphingosine-1-phosphate (S1P), which is abundantly produced by red blood cells, activated platelets, and endothelial cells [50]. Currently S1P is extensively studied and represents a “hot” topic in HSC mobilization [51]. There is evidence that HSC possess receptors for S1P. Through interaction with its receptors, it may control the chemotaxis of HSC between BM, blood, and tissues. Characteristically, it increases in blood and decreases in BM during mobilization, while it also inhibits SDF-1 through the p38/Akt/mTOR pathway [51]. Experimental elimination of S1P_1_ receptors leads to impaired mobilization after CXCR4 inhibitors in mice, while intravenous administration of S1P_1_ agonists induces the opposite effect [52, 53]. The motility of HSC depends on the equilibrium between S1P levels in the circulation and SDF-1 in the bone marrow. While SDF-1 results in the adherence of HSC in the niche, S1P is a major chemoattractant in the blood. This balance is directed towards the egress of HSC to the blood during stress. SDF-1 and S1P are both regulated by specificity-protein-1 (SP1). SP1 acts as a circadian-regulated transcription factor of SDF-1, but it also induces the biosynthesis of S1P [54]. Thus both molecules, which act antagonistically, are closely regulated. Apart from sphingolipids, endocannabinoids induce decreased expression of various adhesion molecules and CXCR4, leading to synergy with G-CSF mobilization [55]. On the contrary, prostaglandin E_2_ (PGE_2_) regulates the expression of CXCR4 on HSC and aids SDF-1 in attracting them to the BM [56].

### 4.4. Adhesion Molecules

Adhesion molecules are surface antigens that facilitate cell-cell and cell-extracellular matrix (ECM) interactions, through their respective receptors. They play a major role in inflammation, mediating trafficking, endothelial rolling, adhesion, and extravasation of leukocytes and lymphocytes. They are essential for cellular immune responses, normal hematopoiesis, and differentiation, as well as for the organization of cells within tissues during ontogenesis. They are widely distributed on hematopoietic and nonhematopoietic cells [57]. According to their structure, they are classified in integrins, molecules belonging to the immunoglobulin superfamily, and selectins. HSC express several types of adhesion molecules, responsible for their interaction with bone marrow stroma.

- The VLA-4/VCAM-1 axis: the *β*1 integrin very late antigen-4 (VLA-4) is expressed by HSC and facilitates their adhesion on BM stroma through interaction with vascular cellular adhesion molecule-1 (VCAM-1), fibronectin, and OPN. Studies in mice, primates, and humans have shown that the administration of the anti-VLA-4 monoclonal antibody (natalizumab) or blockade of its ligands leads to a potent mobilization effect [23, 28, 58]. However, the disruption of the VLA-4/VCAM-1 axis is induced by both G-CSF and CXCR4 inhibitors. Thus it cannot be viewed as an independent/primary mobilization mechanism.
- CD44 (HCAM): CD44 has many isoforms. Among these, CD44s, the smallest one, is the most common one expressed on HSC. The major ligand of CD44 is hyaluronic acid (HA), a component of ECM. The most HA-rich regions of the bone marrow stroma are the endosteum and the sinusoidal endothelium, the sites that are considered the niches of the most primitive HSC. CD44/HA interactions are essential for homing of HSC in the BM. Membranal CD44 cleavage on HSC is associated with mobilization and depends on the equilibrium of a membrane-bound proteolytic enzyme (membrane-type-1 metalloproteinase-MT-1 MMP) and its inhibitor RECK. G-CSF administration leads to increased expression of MT-1 MMP on CD34^+^ cells, which in turn results in membranal CD44 cleavage and HSC egress from the BM [59, 60]. Homing of HSC is impaired after the administration of anti-CD44 antibodies in mice, highlighting its crucial role for SDF-1/CXCR4 binding [60].

### 4.5. SCF/c-kit

Stem cell factor (SCF) is an important chemokine produced by SNO and CAR. Its active form binds to c-kit on the surface of HSC. C-kit (CD117) is a type III tyrosine kinase receptor and is also expressed by endothelial progenitor cells and MSC. The SCF/c-kit axis plays an important role in embryonic hematopoiesis and cellular differentiation. SCF has been shown to augment engraftment in mice through increased CXCR-4 expression on HSC [44]. Experimental elimination of c-kit in mice mobilized with plerixafor or anti-VLA-4 resulted in a negative effect of mobilization [61]. In these c-kit-mutant mice, c-kit activity is defective, but c-kit levels and its binding ability to SCF are normal. Thus the c-kit kinase activity appeared to be crucial for mobilization [61]. However its exact role has not been elucidated, and conflicting results have been reported. In another experiment, the functional blockade of c-kit with a neutralizing antibody was associated with HSC migration from the BM to the blood [62].

### 4.6. Osteoblasts/Osteoclasts (OB/OC)

It is nowadays widely accepted that bone tissue plays an important role in maintaining and regulating the HSC niche. Bone remodeling is a complex process and its consequences on HSC retention and mobilization are an evolving field [63].

Receptor activator of NF-*κ*B ligand (RANKL) is a cytokine that belongs to the tumor necrosis factor superfamily. It is produced by OB, other stromal cells, and activated T-lymphocytes. It stimulates osteoclastic differentiation, maturation, and activation. RANKL upon binding to its receptor RANK (on OC) stimulates osteoclastic activity. RANKL may also bind to osteoprotegerin (OPG). OPG is also produced by OB and acts as a soluble decoy receptor of RANKL that prevents the latter from binding to RANK. Thus the regulation of osteoclastic activation is dependent on RANKL/OPG balance.

The administration of RANKL in mice resulted in an expected increase of osteoclastic activity, but also a simultaneous HSC mobilization, was observed [64]. During the same experiment, the inhibition of OC by exogenous calcitonin led to a decrease of HSC egress from the BM. Thus, it has been proposed that OC produce proteolytic enzymes, such as MMP-9 and cathepsin K, which disrupt important HSC/niche interactions, promoting the release of HSC from the BM [63]. G-CSF has been shown to induce reduction of the number and function of endosteal OB leading to reduced SDF-1 transcription, while it causes an increase of activated osteoclasts [63]. However, experimental data in humans indicate that the augmentation of osteoclastic activity and bone resorption occur a few days later than the typical peak of 4-5 days after G-CSF mobilization [65]. Moreover, inhibition of OC through the administration of bisphosphonates or RANKL neutralizing antibodies does not negatively affect the mobilization of HSC in mice [7]. In line with these findings, osteoporotic mice show impaired HSC mobilization, while increased bone mass is associated with an increase in the HSC pool [66]. Thus the role of OB/OC interactions in regulating mobilization seems more complicated and remains enigmatic.

In an effort to elucidate the role of bone tissue in human HSC mobilization, we undertook a study of various biochemical markers of bone remodeling, osteoclast/osteoblast regulators, and angiogenic cytokines in the process of HSC mobilization. We studied 24 patients, 10 with multiple myeloma, 5 with Hodgkin's Lymphoma, and 9 with non-Hodgkin's lymphoma who were mobilized with G-CSF +/− chemotherapy [67]. We found that both soluble RANKL and OPG increased significantly between premobilization and HSC collection, but the increase in sRANKL was more prominent than OPG, leading to an increased sRANKL/OPG ratio, indicating stimulation of osteoclastic activity during mobilization. However, impressively, we did not observe a respective increase in bone resorption. On the contrary we found a significant increase of bone specific alkaline phosphatase (bALP), which is considered the most sensitive marker of bone formation. In parallel we showed that the ratio of angiopoietin-1/angiopoietin-2 was significantly reduced indicating vessel destabilization. Our results suggest that increased osteoblastic activity and endothelial vessel destabilization are the two major events during human HSC mobilization. Osteoblasts are the orchestrating cells, while osteoclasts are stimulated, but not fully active. Moreover, high levels of bone resorption markers (C-terminal and N-terminal telopeptides) as well as low levels of angiopoietin-1 before the initiation of mobilization are reliable predictors of poor HSC collection.

### 4.7. CD45

CD45, known as leukocyte common antigen, is expressed by all leukocytes, including HSC. It is a transmembrane protein tyrosine phosphatase. Through dephosphorylation of Src kinase, it is involved in signal transduction pathways, regulating several cell processes. CD45 has been mainly studied in lymphocyte maturation, proliferation, and activation. Recent data have demonstrated a pivotal role of CD45 in HSC motility and microenvironmental regulation in both mice and human. From CD45 knockout mice experiments and CD45 treated human HSC in xenotransplantation experiments, it has been shown that CD45 is essential for HSC mobilization. G-CSF leads to increased expression of CD45 in bone marrow mononuclear cells, which in turn correlates with HSC release from the bone marrow. HSC from mice lacking CD45 show reduced mobilization, impaired ability to cross extracellular matrix barriers, and a hyperadhesive phenotype. In addition CD45 is required for normal osteoclastic development and function. CD45 negative osteoclasts display abnormal morphology and have a reduced ability to form multinucleated cells. Moreover they secrete lower amounts of MMP-9 and have a reduced bone resorbing activity. CD45 negative osteoclasts display a reduced response to RANKL resulting in poor HSC mobilization. Thus CD45 is a paradigm of the concurrent regulation of hematopoiesis and the microenvironment [68, 69].

### 4.8. Hypoxia

The main regulator of hypoxia in the endosteal niche is HIF-1, consisting of HIF-1*α* and HIF-1*β*. Under conditions of adequate oxygenation, HIF-1*α* is hydroxylated and is recognized by Von Hippel-Lindau protein, leading to its proteasome-mediated degradation [70]. Under hypoxic conditions (which are prevalent during G-CSF mobilization), the hydroxylation of HIF-1*α* is prevented, and HIF-1*α* is stabilized. The full heterodimeric HIF-1 binds to hypoxia response elements of the genome, resulting in the transcription of several genes, including VEGFA in the BM sinusoids [71]. VEGF results in vasodilatation and HSC mobilization.

It has been shown that activation of m-TOR and the resultant increase of reactive oxygen species (ROS) in the BM lead to the egress of HSC, whereas the inhibition of mTOR with rapamycin has the opposite effect. It appears that a “critical” level of ROS and HIF-1*α* are necessary for mobilization [72].

## 5. Clinical Applications of HSC Mobilization Mechanisms

As already stated, the main initiating molecule of the HSC mobilization process is exogenous or chemotherapy-induced endogenous G-CSF. For many years, the administration of G-CSF with or without chemotherapy has been the mainstay of mobilization in every day clinical practice. Ifosfamide-containing (IGEV and ICE) or platinum-containing (ESHAP and DHAP) regimens with G-CSF are usually applied in lymphomas [73–75]. These regimens have a triple role: they offer disease control, they test tumor chemosensitivity, and they result in excellent HSC mobilization. In multiple myeloma, either cyclophosphamide at high or intermediate doses in combination with G-CSF or G-CSF alone may be used [76].

The endpoint of every mobilization attempt is the collection of adequate numbers of HSC, reflected practically by the absolute number of CD34^+^ cells collected per kg of body weight. Adequate number is considered the dose of HSC that ensures rapid and sustained long-term hematopoiesis after the administration of myeloablative chemotherapy and HSC infusion. The optimal dose of HSC is 5 × 10^6^/kg, with little clinical benefit with doses 5–8 × 10^6^/kg and no further improvement with grafts containing >10 × 10^6^/kg CD34^+^ cells [77]. The lowest acceptable dose is 2 × 10^6^/kg.

Still, there are a substantial proportion of patients who fail to mobilize and collect adequate numbers of HSC and consequently cannot proceed to autologous stem cell transplantation. The inability of adequate HSC mobilization is associated with HSC reserve exhaustion and disruption of BM stroma [78]. However, even 5% of healthy donors fail to mobilize adequate numbers of HSC. Genetic polymorphisms in molecules associated with the BM niche, such as VCAM-1 and SDF-1, have been implicated in this [79].

Risk factors for poor mobilization include age >65 years, bone marrow cellularity <30%, bone marrow infiltration, multiple previous chemotherapy regimens, especially alkylating agents, fludarabine, >4 cycles of lenalidomide, and previous extended field radiotherapy including the pelvis [80, 81].

There is not a widely accepted definition of the “poor mobilizer.” For this purpose, the Italian group of apheresis has proposed criteria not only for the definition, but also for the early recognition of the poor mobilizer [81]. According to these criteria a patient is considered as a proven poor mobilizer if he/she collected <2 × 10^6^/kg CD34^+^ cells through three consecutive days of apheresis, after the administration of an adequate mobilization regimen (G-CSF monotherapy at a dose of ≥10 *μ*g/kg/day or chemotherapy + G-CSF ≥ 5 *μ*g/kg/day) [82]. However patients who fail to achieve a maximum peak of circulating CD34^+^ cells < 20/*μ*L on the day of the expected peak are also considered poor mobilizers [83]. The day of the expected peak is dependent on the mobilizing regimen. Thus with G-SCF monotherapy at a dose of 10 *μ*g/kg, peak levels of CD34^+^ cells are expected on the 5th-6th day after the initiation of G-CSF. After ifosfamide-containing regimens + G-CSF, the peak day is usually the 12th-13th day since chemotherapy initiation, whereas the corresponding day for platinum-containing chemotherapy ranges between the 15th–17th day.

## 6. Plerixafor in HSC Mobilization

Plerixafor is a reversible CXCR4 antagonist and its mechanisms of action have been already described. It has been approved in both the US and the EU for the mobilization of patients with lymphoma and myeloma. With its use in combination with G-CSF, approximately 70% of poor mobilizers may succeed in the collection of adequate numbers of HSC in order to proceed to ASCT [84]. The superiority of plerixafor in combination with G-CSF over G-CSF alone was established by several phase II [85–89] and two phase III multicenter randomized double-blinded placebo controlled studies [84, 90]. In the lymphoma trial [84] a significantly higher proportion of patients (59.3%) achieved optimal CD34^+^ cell collection (≥5 × 10^6^/kg) in the G-CSF plus plerixafor arm compared to the G-CSF plus placebo arm (19.6%). In addition 86.7% of the patients mobilized with G-CSF plus plerixafor reached the minimum target of ≥2 × 10^6^/kg CD34^+^ cells versus 47.3% of those who received G-CSF plus placebo. The number of apheresis sessions was also significantly lower in the combination arm. The median fold increase in circulating CD34^+^ cell count from day 4 to day 5 was 5.0 in the plerixafor treated patients and 1.4 in the placebo treated patients. Similarly in the myeloma study [90] 71.6% of the G-CSF plus plerixafor treated patients collected ≥6 × 10^6^/kg CD34^+^ cells in ≤2 apheresis procedures versus 34.4% of the G-CSF plus placebo group. Plerixafor has an excellent safety and tolerability profile.

The high cost associated with plerixafor requires a stringent protocol with well-defined criteria for its proper use [84, 88, 91]. Plerixafor can be applied with three strategies: (i) identification of patients with risk factors for poor mobilization and upfront use of plerixafor. This strategy is still outside the current approved indications and is not recommended; (ii) combination of G-CSF and plerixafor in a second mobilization attempt for patients who have already failed a prior HSC collection. This is the current approved usage of the drug; G-CSF is given at a dose of 10 *μ*g/kg/day × 4 days and plerixafor is administered at a dose of 240 *μ*g/kg on the fourth day, 9–11 hours before collection on the 5th day; however with this strategy, significant time is lost between the mobilization attempts, which might be crucial for some patients with aggressive disease; in addition, the repetition of the whole procedure is cumbersome for the personnel and inconvenient for the patient; (iii) the preemptive—“just in time”—use of plerixafor seems to be the most cost-effective strategy: the proper mobilization regimen according to the patient's disease status is chosen. If CD34 counts in peripheral blood on the day of the expected peak are suboptimal (<20/*μ*L), plerixafor is administered prior to HSC collection. With this latter strategy >90% of the patients can achieve grafts with >2 × 10^6^/kg CD34^+^ cells [92]. Another practical issue with plerixafor is the optimal timing of its administration. Current data indicate that the peak of circulating CD34^+^ cells can be achieved in 3–8 hours after its administration, which may be more convenient for organizing the procedure of apheresis [93].

Although plerixafor rescues a significant proportion of patients from failing collection of HSC, there is still a 35% possibility of its failure. Towards this aim, new mobilizing agents are being tested either in preclinical or clinical studies.

## 7. Novel Agents Tested in Human Clinical Studies

- Agents targeting the SDF1/CXCR4 axis: POL6326 (Polyphor, Allschwil, Switzerland) is a potent selective CXCR4 inhibitor, which has shown considerable efficacy as monotherapy in newly diagnosed multiple myeloma patients [94]. Its excellent safety and tolerability profile renders it a promising agent and is currently tested in further dose escalation. BKT140 (Biokine Therapeutics, Rehovot, Israel) is another promising high affinity CXCR4 inhibitor that has been tested in a phase I/IIa trial in multiple myeloma patients in combination with intermediate dose cyclophosphamide and G-CSF. BKT140 resulted in a significant collection success that reached a mean absolute of 20.6 × 10^6^/kg CD34^+^ collected cells for the highest dose used. Moreover the number of apheresis procedures was reduced with the highest doses. This agent warrants further evaluation, since it also shows antimyeloma activity [95, 96]. Other molecules belonging to this category are TG-0054 (Taigen Biotechnology, Taipei, Taiwan) [97] and NOX-A12 (NOXXON Pharma, Berlin, Germany) [98]. The latter is a structured mirror-image RNA oligonucleotide, a so-called Spiegelmer that binds SDF-1, thereby inhibiting its activity.
- Proteasome inhibitors: bortezomib is nowadays considered among the leading therapeutic drugs in multiple myeloma. Surprisingly, in animal studies, bortezomib proved to be a potent mobilizing agent, increasing significantly CFU-Cs compared to placebo. In addition, a synergistic effect of bortezomib with G-CSF, plerixafor, and chemotherapy was evident [99]. The most likely mobilizing mechanism of bortezomib is the disruption of VLA-4/VCAM-1 axis. In a recent phase II study, the addition of bortezomib to cyclophosphamide and G-CSF during mobilization resulted in a median CD34^+^ yield of 23.2 × 10^6^/kg in a median of 1 apheresis session [100].
- Parathormone (PTH): Brunner et al. showed that PTH induces a significant increase of progenitor cells in the peripheral blood (1.5- to 9.8-fold) of mice. The authors postulated that this activity relates to endogenous release of G-CSF [101]. PTH was evaluated in a phase 1 study in escalating doses in combination with G-CSF in patients who had failed prior mobilization attempts: 47% and 40% of the patients who had failed one and two prior mobilization attempts, respectively, succeeded in reaching the mobilization criteria of the study [102].

## 8. Novel Agents Evaluated in Animal Studies

- *VLA-4/VCAM-1 Inhibitors*. The importance of VLA-4/VCAM-1 axis has been already extensively described. Papayannopoulou and Nakamoto were among the pioneers in studying the effect of the VLA-4/VCAM-1 axis disruption in HSC mobilization. They showed that the administration of anti-VLA-4 antibodies resulted in the mobilization of HSC into the circulation [103]. Moreover, the concurrent inhibition of VLA-4 and CXCR-4 has a synergistic mobilization effect in primates [104]. Although, anti-VLA-4 antibodies have not been tested in human mobilization, there are data indicating that it has a mobilizing effect in the human as well. Natalizumab is a humanized monoclonal antibody against the *α*4 subunit of VLA-4, approved for treatment of multiple sclerosis (MS) and Crohn's disease. In patients with MS treated with Natalizumab, a gradual increase in circulating CD34^+^ cells has been observed with a peak being present in 3-4 days after treatment. However the persistence of CD34^+^ cells in the circulation for an extended time period may be problematic in clinical practice [58, 105]. The study of other small inhibitors of the VLA-4/VCAM-1 axis is ongoing.
- Other novel agents currently evaluated in preclinical studies are FG-4497 that stabilizes HIF-1 through inhibition of its hydroxylation [106], Gro*β* (CXCL2), a chemokine whose exact mechanism is unclear [107], and S1P agonists [52].

## 9. Conclusions

The mechanisms of HSC mobilization are overlapping and not fully elucidated yet. Experimental studies have given light into many aspects of this cascade of events. However, we are still far away from establishing an integrated model of the mechanisms that control the equilibrium of HSC between quiescence and mobilization. Laborious research is mandatory for the development of newer agents that might render HSC mobilization and collection possible for all patients.

## Figures and Tables

**Table 1:** Receptors, ligands, and adhesions molecules involved in the homeostasis of the HSC niche.

Expression from HSC		Expression from BM stroma
Tie2	⟺	Ang-1
Mpl	⟺	TPO
c-kit (CD117)	⟺	SCF
CXCR4	⟺	SDF-1 (CXCL12)
TGF-*β*R	⟺	TGF-*β*
FGFR1-4	⟺	FGF
Notch	⟺	Jagged-1
GRP78	⟺	Cripto
		PTH-R
HIF-1*α*	⟺	Cripto
N-cadherin	⟺	N-cadherin
BMP-R2A	⟺	BMP
VLA4	⟺	VCAM-1
Frizzled	⟺	Wnt (ECM)
FLT-3 (CD135)	⟺	FLT3 ligand
CaR	⟺	Ca^++^ (ECM)
HCAM (CD44)	⟺	Hyaluronan (ECM)
LFA-1	⟺	ICAM-1
VEGF	⟺	VEGFR
Agrin-R	⟺	Agrin proteoglycan (ECM)
S1P_1_	⟺	S1P

Ang-1: angiopoietin-1, SCF: stem cell factor, SDF-1: stromal-derived factor-1, FGF: fibroblast growth factor, PTH-R: parathormone receptor, Cripto: teratocarcinoma derived growth factor-1/TDGF-1, BMP: bone morphogenic protein, VLA4: very late antigen 4, VCAM-1: vascular cellular adhesion molecule-1, FLT-3: fms-like tyrosine kinase 3, HCAM: hyaluronan binding-cellular adhesion molecule, LFA-1: lymphocyte function-associated antigen-1, VEGF: vascular endothelial growth factor, S1P: sphingosine-1-phosphate, and ECM: extracellular matrix.
